# Respectful maternity care and its relationship with childbirth experience in Iranian women: a prospective cohort study

**DOI:** 10.1186/s12884-020-03118-0

**Published:** 2020-08-17

**Authors:** Khadije Hajizadeh, Maryam Vaezi, Shahla Meedya, Sakineh Mohammad Alizadeh Charandabi, Mojgan Mirghafourvand

**Affiliations:** 1 Midwifery Students’ Research Committee, Midwifery Department, Tabriz University of Medical sciences, Tabriz, Iran; 2 Fellowship of gynecology oncology, Alzahra teaching hospital, Tabriz University of Medical Sciences, Tabriz, Iran; 3 School of Nursing, Faculty of Science, Medicine and Health, University of Wollongong, Wollongong, Australia; 4 Department of Midwifery, Faculty of Nursing and Midwifery, Tabriz University of Medical Sciences, Tabriz, Iran; 5 Social Determinants of Health Research Center, Tabriz University of Medical Sciences, Tabriz, Iran

**Keywords:** Respectful maternity care, Birth experience, Prospective cohort study, Iran

## Abstract

**Background:**

Intrapartum respectful maternity care is defined as a fundamental human right that can affect the mother’s experiences. This study aimed to determine the status of respectful maternity care and its relationship with childbirth experience among Iranian women.

**Methods:**

This prospective cohort study recruited 334 postpartum women in postpartum wards of two public and four private hospitals in Tabriz, Iran. Quota sampling was used based on the number of births in each hospital. Data were collected through interviews with the use of the following tools: sociodemographic and obstetrics characteristics questionnaire, respectful maternity care scale (6 to 18 h postpartum), and childbirth experience questionnaire (30 to 45 days postpartum). The General Linear Model was used to determine the relationship between respectful maternity care and childbirth experience.

**Results:**

The mean respectful maternity care score was 62.58 with a range of 15 to 75, and the total childbirth experience score was 3.29 with a range of 1 to 4. After adjusting for sociodemographic and obstetrics characteristics, a statistically significant direct correlation was found between respectful maternity care and a positive childbirth experience (*P* < 0.001).

**Conclusions:**

The findings reveals a direct relationship between respectful maternity care and positive childbirth experience. Therefore, it is recommended that mangers and policy makers in childbirth facilities reinforce facilitating a respectful maternity care to improve women’s child birth experience and prevent potential adverse effects of negative childbirth experiences.

## Background

Respectful maternity care (RMC) has been promoted in recent years due to the importance of ethical, psychological, social, and cultural aspects of childbirth among different populations [1]. Although there is no consensus on the respectful maternity care definition, it is usually regarded synonymous with friendly and woman-centered care. Intrapartum respectful maternity care is defined as a fundamental human right that includes respecting women’s beliefs, independence, emotions, dignity, and preferences to reserve their right of having a companion or performing their cultural rituals [2, 3]. Disrespect and abuse (D&A) violate the basic principles of ethics, human rights, and basic obligations in providing care for patients [4]. D&A is sometimes exacerbated in a way that caring for patients at a health center becomes more dangerous than caring for them at home, as it can cause a severely negative experience for the mother during childbirth [5].

Traumatic childbirth experience varies from country to country (the Netherlands 0.16% [6], Sweden 0.7% [7], Norway 21% [8] and Iran 37% [9]). Different intrapartum factors can affect the experience of traumatic childbirth. For example, fear of childbirth, failure to take analgesics [9], lack of support [10], and transferring the newborn to the Neonatal Intensive Care Unit (NICU) [11] can be among risk factors for traumatic childbirth. In a cross sectional study among 800 Iranian women, absence of pain relief during labour and the fear of childbirth were the main intrapartum predicting factors for a traumatic birth experience [9].

Traumatic childbirth experience can have many negative effects, including poor mother-baby bond, unwillingness to breastfeed [12], PTSD [13], and poor quality of life [14]. A study aiming to explore the impact of D&A on women’s experiences in Denmark showed that childbirth abuse can subsequently affect women’s reproductive health patterns including sexual relationship, desire for subsequent pregnancy and making decisions regarding the type of childbirth undergone (vaginal delivery or cesarean section) [15]. D&A experiences in the delivery room can also be a deterrent to the future use of healthcare services [16, 17].

Although these negative experiences can fade over time, they may persist in women’s memory for up to 5 years [18]. The WHO has also advised respectful maternity care in a statement on recommendations to improve childbirth experience. These recommendations are emphasized in the context of effective communications between caregivers and mothers, participation in selection and continuity of care [19].

Disrespectful care and abuse has been studied in many countries [20–22], however, based on our knowledge, there is no Iranian study that assessed the respectful maternity care status and its relationship with childbirth experience. Therefore, the present study aimed to explore the relationship between respectful maternity care and childbirth experience among a group of Iranian women.

### Ethical consideration

This study was approved by ethics committee of Tabriz University of Medical Sciences (Ethical code: IR.TBZMED.REC.1398.202). A written informed consent was obtained from the participants. Ethical considerations including women’s confidentiality, privacy, and voluntary participation were respected in this study.

## Methods

The present study is the first phase of a mixed-method study conducted to develop guidelines to facilitate improving respectful maternity care in hospitals [23]. The first phase was a prospective cohort study that enrolled 334 postpartum women who gave birth in public and private hospitals in the city of Tabriz. The STROBE checklist was used to report this study.

### Study participants

Women who lived in Tabriz and had vaginal birth with no infant death or major malformations were included in the study. Women who were deaf or mute or had a history of depression, other mental health disorders, stressful events such as divorce, death of a first-degree family member, diagnosis of incurable or hard-to-treat diseases in a family member three months before the study were excluded from the study.

### Sample size

This study is part of a larger study in which [23] the sample size of 334 was estimated using a one-sample proportion estimation formula based on 32% prevalence rate of D&A in an Ethiopian study[24], d = 0.05, Z = 1.96 and q = 0.68.

### Recruitment and Sampling

Postnatal women from the maternity wards who met the criteria were invited to participate in the study. If the potential participants met the inclusion criteria, they were briefed on the objectives and methods of the study and they were asked to sign written informed consent if they were willing to participate in the study. A quota sampling was used based on the number of childbirths three months prior to the study in each study setting. The study settings included two public hospitals (Alzahra and Taleghani) and four private hospitals (29 Bahman, Behbood, Nor-e-Nejat, and Shahriyar) in Tabriz-Iran.

### Data collection

The data were collected at two points: the recruitment point (6 to 18 h postpartum) and in the following interview (30 to 45 days after birth). The following tools were used for data collection: sociodemographic and obstetrics characteristics questionnaire, and respectful maternity care scale, and childbirth experience questionnaire. The language used in the questionnaire was Farsi.

### Data collection tools

Sociodemographic and obstetrics characteristics questionnaire was developed by the research team. Face and content validities were used to assess the validity of the tool. The questionnaire was then distributed to 10 faculty members of Tabriz University of Medical Sciences. The research team made the necessary corrections based on the feedback received. The questions included four main categories: (a) socio-demographic factors (age, education level, occupation, source of support, marital satisfaction, husband’s education); (b) pregnancy-related factors (planned pregnancy, history of abortion); (c) intrapartum factors (place and time of birth, duration of labour, gestational age, augmentation, birth attendant, length of stay in the labour room, number of healthcare providers); (d) neonatal factors (sex, admission to Neonatal Intensive Care Unit ).

The scale that was used to measure respectful maternity care was developed initially by sheferaw et al. (2016) in Ethiopia demonstrating its validity and reliability ((α = 0.845) [25]. This instrument consists of four domains and 15 items: friendly care (7 items), abuse-free care (3 items), timely care (3 items), and discrimination-free care (2 items). The responses are as follows: strongly agree (score 5), agree (score 4), no comments (score 3), disagree (score 2), and strongly disagree (score 1). Statements with negative conceptions were scored negatively. High mean scores on this scale indicate a more positive respectful maternity care experience during childbirth [25].The validity and reliability of respectful maternity care scale in Farsi language has been accessed in another project (the paper is under review). In our study, Cronbach’s alpha coefficient was obtained as 0.93 and intraclass correlation coefficient (ICC) (95% confidence interval) was 0.98 (0.96 to 0.99).

Childbirth Experience Questionnaire (CEQ2) that was developed by Dencker et al. (2010) [26] was used to measure women’s childbirth experience. CEQ2 consists of 4 domains consists with 22 statements: (a) own capacity (sense of control, personal feeling about childbirth and labor pain), (b) professional support (midwifery information and care), (c) perceived safety (feeling safe and childbirth memories), and (d) participation (individual ability to change posture, movements and pain relief during labor and delivery). Nineteen statements are in form of 4-option items and 3 statements are in the visual assessment (VAS) form the responses are strongly agree (score 1), often agree (score 2), often disagree (score 3), and strongly disagree (score 4). Items answered in form of VAS are converted to values ​​of 1 to 4: Scores of 0–40 (score 1), scores of 41–60 (score 2), scores of 61–80 (score 3) and scores of 80–100 (score 4). Statements with negative concepts were negatively scored. Higher mean scores of this tool means a more positive childbirth experience [26]. Although the validity and reliability of the original tool has been proven among American population, the psychometric evaluation of this tool among Iranian population was also confirmed by Ghanbari et al. (2019) with the Cronbach’s alpha coefficient of 0.93 and ICC of 0.97 [27].

### Data analysis

SPSS version 24 was used for data analysis. The data obtained from sociodemographic, obstetrics characteristics and respectful maternity care questionnaires were described with descriptive statistics of frequency (percent) and mean (standard deviation). Pearson correlation test was used to determine the relationship between respectful maternity care score and childbirth experience score in bivariate analysis. General linear model (GLM) was used in multivariate analysis after controlling the sociodemographic and obstetrics variables. P < 0.05 was taken as significant. Subgroup analysis was also performed for participants from private vs. public hospitals.

## Results

### Participants’ characteristics

A total of 358 mothers assessed for the eligibility and 334 women entered into the study from June 10 to September 1, 2019. All the mothers were followed up in the study with no drop out. About half of the mothers (48.5%) were aged 26–35 years and had a high school education (43.7%). The majority of mothers (95.5%) were housewives with a moderate economic status (76.6%). Nearly half (43.7%) of the mothers stayed less than 5 h in the hospital while their mothers or fathers were in the waiting room helping them as their support people (48.8%). (Table 1).

**Table 1:** Socio-demographic, pregnancy characteristics among postpartum women (*n* = 334)

Variables	n %		Variables	n %	Variables	n %
**Age** (Years)		**Economic status**			**Length of stay in labour** (Hour)	
18–25	132 (39.5)	Low		24 (7.2)	5 and below	146 (43.7)
26–35	161 (48.5)	Moderate		256 (76.6)	6–10	109 (32.6)
36 and above	41 (12.3)	High		54 (16.2)	11 and above	79 (23.6)
**Work status**		**Husband’s education**			**Gestational age at childbirth** (week)	
Homemaker		Elementary and lower		65 (19.5)	Lower than 38	79 (23.7)
Employed	319 (95.5)	Intermediate		72 (21.6)	38 and higher	255 (76.3)
**Education**	15 (4.5)	High school		132 (39.5)	**Gravid**	
Elementary and lower	58 (17.4)	University		65 (19.5)	One	141 (42.2)
Intermediate	79 (23.7)	**Number of healthcare providers**			Two	113 (33.8)
High school	146 (43.7)	One		42 (12.6)	Three	55 (16.5)
University	51 (15.3)	Two		77 (53.0)	Four and above	25 (7.5)
**Husband’s age** (Years)		3 and above		115 (34.4)	**Abortion history**	
18–25	38 (11.4)	**Birth attendant**			No abortion	259 (77.5)
26–35	196 (58.7)	Midwife		77(23.1)	One	60 (18.0)
36 and above	100 (29.9)	Obstetrician (resident or on call)		199 (59.6)	Two and above	15 (4.5)
**Husband’s Job**		Student (midwifery or intern)		16 (4.8)	**Marital satisfaction**	300 (98.8)
Unemployed	12 (3.6)	Personal physician or midwife		42 (12.6)	**Delivery time** (Day)	192 (57.5)
Employed	20 (6.0)	**Planed pregnancy**		221 (66.2)	**Baby sex** (Girl)	161 (48.2)
Self-employed	117 (35.0)	**Use of the pain relief**		101 (30.2)	**Source of support**	
Manual worker	185 (55.4)	**Admission to NICU**		47 (14.1)	Husband	27 (8.1)
**Child birth at Public hospital**	298 (89.2)	**Augmentation for labor**		144 (48.3)	Mother or father	163 (48.8)
		**Duration of labour**		37 (11.1)	Relative	144 (43.1)

### Status of respectful maternity care and childbirth experience

The mean (SD) respectful maternity care score was 62.58 (12.1) with a range of 15 to 75. For consistency in the report, the scores were converted to the sale of 0 to100. Thus, the mean (SD) RESPECTFUL MATENITY CARE score was 63.42 (19.28) in total. The four respectful maternity care subdomain scores were: (a) friendly care 63.57 (19.28), (b) abuse-free care 65.28 (17.74), (c) timely care 57.76 (13.93), and (d) discrimination-free care 65.89 (16.98).

The mean (SD) overall score of childbirth experience was 3.29 (0.13) with the subscales as follows: own capacity 3.44 (0.13), participation 3.21 (0.13), perceived safety 3.05 (0.13) and professional support 3.45 (0.13) of the score range of 1–4 (Table 2).

### The relationship between respectful maternity care and childbirth experience

According to the Pearson correlation test, there was a significant relationship between total score of childbirth experience and the total score of respectful maternity care (*r* = 0.85, P < 0.001) and its subscales with overall childbirth experience score including: (a) friendly care (*r* = 0.82, *P* < 0.001), (b) abuse-free care (*r* = 0.77, *P* < 0.001), (c) timely care (*r* = 0.68, *P* < 0.001), and (d) discrimination-free care (*r* = 0.77, *P* < 0.001) (Table 2).

**Table 2:** The Mean (SD) of the Respectful Maternity Care (RMC) and birth experience (*n*=334)

Variable	Mean (SD^b^)	Mean (SD) of 0 to 100	Obtainable Score Range	Obtained Score Range	Relationship with birth experience r (*P*-value)^a^
**Total score of RMC**	62.58 (12.1)	63.42 (19.2)	15–75	20–74	0.001) > 0.85 (P
Friendly care	29.25 (6.74)	63.57 (19.2)	7–35	7–35	0.001) > 0.82 (P
Abuse free care	12.77 (2.66)	65.28 (17.7)	3–15	4–15	0.001) > 0.77 (P
Timely care	11.66 (2.08)	57.76 (13.9)	3–15	3–15	0.001) > 0.68 (P
Discrimination free care	8.85 (1.69)	68.59 (16.98)	2–10	2–10	0.001) > 0.77 (P
**Total score of birth experience**	3.29 (0.13)	----	1–4	1–4	-----
Own capacity	3.44 (0.13)	-----	1–4	1–4	----
Participation	3.21 (0.13)	-----	1–4	1–4	----
Perceived safety	3.05 (0.13)	-----	1–4	1–4	----
Professional support	3.45 (0.13)	-----	1–4	1–4	----

^a^Standard Deviation, ^b^Pearson correlation test

**Fig. 1:**
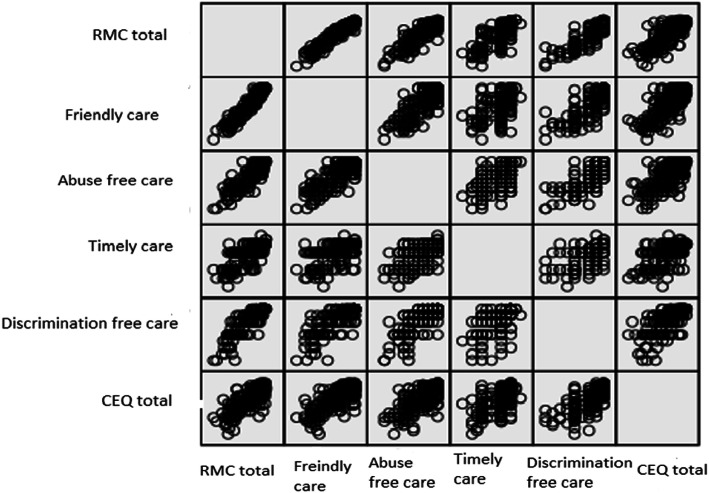
Correlation between respectful maternity care and childbirth experience

The results of subgroup analysis based on private vs. public hospitals showed that there was a significant relationship between childbirth experience and the total score of respectful maternity care and its subscales in both public and private hospitals (*P* < 0.05) except for friendly care domain in private hospitals (*P* = 0.053) (Table 3). Figure 1 shows the strong positive correlation between respectful maternity care and positive childbirth experience.

**Table 3:** The Mean (SD) of the Respectful Maternity Care (RMC) and birth experience based on hospital type

Variable	Public hospital (*n* = 298)		Private hospital (*n* = 36)	
	Mean (SD^b^)	Relationship with birth experience r (*P*-value)^a^	Mean (SD^b^)	Relationship with birth experience r (*P*-value)^a^
**Total score of RMC**	61.50 (12.44)	0.85 (< 0.001)	71.38 (3.24)	0.56 (< 0.001)
Friendly care	28.63 (6.87)	0.82 (< 0.001)	34.33 (1.56)	0.32 (0.053)
Abuse free care	12.57 (2.71)	0.77 (< 0.001)	14.63 (0.83)	0.53 (0.001)
Timely care	11.55 (2.15)	0.68 (< 0.001)	12.55 (1.18)	0.52 (0.001)
Discrimination free care	8.78 (1.75)	0.77 (< 0.001)	9.86 (0.42)	0.58 (< 0.001)

^a^Standard Deviation, ^b^Pearson correlation test

Based on the independent t-test and one-way analysis of variance, there was a statistically significant relationship between childbirth experience and the variables of source of support, marital satisfaction, occupation, time of childbirth, type of hospital, birth attendant, length of stay in labour, number of healthcare providers, duration of labour, gestational age and augmentation of labor (*P* < 0.05). All of these variables were entered into General linear model along with respectful maternity care. After adjusting for sociodemographic and obstetrics characteristics, the results of the General linear model (showed that childbirth experience score increased significantly with an increase in respectful maternity care score (unstandardized coefficients: 0.04, 95% confidence interval: 0.03 to 0.04, *P* < 0.001) (Table 4).

**Table 4:** The Relationship of Respectful Maternity Care (RMC) With Childbirth Experience (CE) Based on the General Linear Model

Variable	B (95% Confidence Interval)	P
**Total score of RMC**	0.04 (0.03 to 0.04)	0.001>
**Childbirth place** (Reference: Private hospital)		
Public hospital	-0.16 (-2.31to 0.19)	0.884
**Delivery time** (Reference: Night)		
Day	-0.04 (-0.12 to 0.03)	0.309
**Duration of labour** (Reference: No)		
Yes	-0.83 (-0.23 to 0.07)	0.296
**Augmentation for labor** (Reference: No)		
Yes	-0.28 (-1.17 to 0.06)	0.544
**Work status** (Reference: Employed)		
Homemaker	0.20 (0.19 to 0.38)	0.030
**Birth attendant** (Reference: Personal physician or midwife )		
Midwife	0.03(-0.17 to 0.24)	0.753
Obstetrician (Resident or on call)	-0.02 (-0.23 to 0.18)	0.828
Student (Midwifery or intern)	0.02 (-0.24 to 0.28)	0.867
**Number of health care providers** (Reference: Three and more)		
One	0.071 (-0.09 to 0.23)	0.395
Two	0.04 (-0.06 to 0.14)	0.450
**Length of stay in labour** ( Reference: 11 h and above)		
5 h and below	-0.68 (-0.21 to 0.07)	0.354
6–10 h	-0.23 (-0.15 to 0.10)	0.723
**Source of support** (Reference: Relatives)		
Husband	-0.03 (-0.18 to 0.10)	0.599
Mother or father	-0.08 (-0.016 to -0.07)	0.032
**Marital satisfaction** (Reference: No)		
Yes	0.06 (-0.02 to 0.14)	0.150

## Discussion

The study aimed to assess the status of respectful maternity care and its relationship with childbirth experience in postpartum women at public and private hospitals in Tabriz, Iran. The mean respectful maternity care score was 63.42 with a range of 0 to 100. The highest respectful maternity care pertained to discrimination-free care subscale and the lowest respectful maternity care pertained to timely care subscale. The mean score of respectful maternity care and its subdomains was significantly related with the mean score of childbirth experience.

### Status of respectful maternity care

In our study in more than half of the women (63.42%) only reported respectful maternity care which is consistent with the findings of a study conducted in Ethiopia (66%) by Sheferaw et al. (2017) [25]. This levels of respectful maternity care in Iran may appear to be due to the fact that childbirth in Iran is not women-centered and mainly focused on medical interventions such as performing episiotomy and routine use of oxytocin without decision making process [9, 28].

Women perceived less respectful maternity care when there was a delayed in providing care or keeping mothers waiting (timely care subdomain). The result of our study is align with the study conducted by Mousa and Turingan (2019) where they reported the lowest respectful maternity care score related to the timely care subscale [29]. These results are also consistent with other studies where delayed care and abandonment of women during childbirth were reported as the most common complaints of women for lack of respectful maternity care [30]. Bohren et al. also reported that women repeatedly mentioned prolonged delay in receiving healthcare personnel attendance in the delivery room [31] as part of poor quality in their care. To address this issue, WHO recommends the provision of midwife-led continuity models of care, in which women are supported by a midwife or a small group of midwives throughout the prepartum, intrapartum, and postpartum periods [32]. Continuity of care in midwifery demonstrated high satisfaction among women towards the care they receive [33]. Continuity in maternity care, education and support is recommended for better maternal and neonatal outcomes [33, 34].

In this study, the highest respectful maternity care score related to discrimination-free care. Our study is consistent with the study by Mousa, where 85% of their participants who reported respectful maternity care, when they received discrimination-free care [29]. In the review study by Bohren, women complained about ethnic and racial discrimination. Discrimination in birthing rooms was related to their ethnicity, race, religion, age, socioeconomic status and medical conditions [31]. The reason women in our study reported a high prevalence of discrimination-free care could be attributed to have consistency in race, ethnicity, religion, nationality, and socioeconomic status of the women who participated in the study. Fear of such discrimination can be an important reason for unwillingness of pregnant women to give birth in delivery facilities [35].

### The relationship between respectful maternity care and childbirth experience

In the present study, high respectful maternity care was associated with positive childbirth experience. Although there is no quantitative study regarding causation effects among these two variables, the findings of several qualitative studies suggest that disrespect and abuse during childbirth impacts women’s childbirth experiences negatively. For example, in a qualitative study by Orpin et al. (2018) in Nigeria postpartum women reported shouting and verbal abuse as a common practice in the delivery room which perceived inhumane and contrary to the human dignity [36]. Two studies in Nigeria and Guinea on women’s birth experiences regarding D&A showed that women do not accept verbal and physical D&A, unless there is a risk for the mother or infant. Interestingly, the care providers also believed that it is acceptable to use verbal abuse and disrespect women when the mother or infant is at risk. Health care providers perceived those behavior justified when the women are not cooperative or obedient for the medical instructions [37, 38]. In our study, it was not explored if women perceived any disrespect and abuse behaviors as part of a normal care.

### Strength and limitation

The main strength of the study is the potential for the generalizability of the findings due to the broad range of population that included primiparous and multiparous women with term, preterm, singleton and twin deliveries from both public and private hospitals. A limitation of the present study is that all the women in our study were from the same city. Therefore, it is suggested that a similar study may be beneficial to conduct in other areas of Iran that includes women from rural areas with different cultural backgrounds and ethnicities.

There were two potential biases in this study including attrition bias and response bias (not reporting of events due to sense of shame and embarrassment or perceiving abusive care as normal care). We minimized the attrition bias through accurate follow-up by phone contacts (twice a week). To minimize the response bias, the interviews were conducted in a private room and the participants were ensured about confidentiality and anonymity.

## Conclusions

Based on the results of this study, direct relationship was observed between respectful maternity care and positive childbirth experience. Because women are vulnerable in any way, during pregnancy, especially during labor and childbirth, therefore, failure to receive respectful maternity care can result in negative experiences including fear of childbirth and reduced vaginal childbirth and a desire to have cesarean section. So, healthcare providers should pay more attention to women during childbirth. Also, it is imperative that policy makers in maternity care adopt solutions to reinforce respectful maternity care behavior in birthing rooms and protect women from any disrespect of abuse. Women also need to be educated about their rights and respectful maternity care principles in childbirth preparation classes.

## Data Availability

The datasets used and analyzed during the current study are available from the corresponding author on reasonable request.

## Abbreviations

D&A: Disrespect and Abuse
RMC: Respectful Maternity Care
WHO: World Health Organization
ICC: Intra-class Correlation Coefficient
SD: Standard Deviation
